# Analysis of meiotic recombination in 22q11.2, a region that frequently undergoes deletions and duplications

**DOI:** 10.1186/1471-2350-8-14

**Published:** 2007-04-02

**Authors:** Laura Torres-Juan, Jordi Rosell, Manuel Sánchez-de-la-Torre, Joan Fibla, Damià Heine-Suñer

**Affiliations:** 1 Secció de Genètica, Hospital Universitari Son Dureta, Andrea Doria 55, Palma de Mallorca 07014, Balears, Spain; 2 Departament de Ciències Mèdiques Bàsiques, Irblleida, Universitat de Lleida, Lleida, Spain

## Abstract

**Background:**

The 22q11.2 deletion syndrome is the most frequent genomic disorder with an estimated frequency of 1/4000 live births. The majority of patients (90%) have the same deletion of 3 Mb (Typically Deleted Region, TDR) that results from aberrant recombination at meiosis between region specific low-copy repeats (LCRs).

**Methods:**

As a first step towards the characterization of recombination rates and breakpoints within the 22q11.2 region we have constructed a high resolution recombination breakpoint map based on pedigree analysis and a population-based historical recombination map based on LD analysis.

**Results:**

Our pedigree map allows the location of recombination breakpoints with a high resolution (potential recombination hotspots), and this approach has led to the identification of 5 breakpoint segments of 50 kb or less (8.6 kb the smallest), that coincide with historical hotspots. It has been suggested that aberrant recombination leading to deletion (and duplication) is caused by low rates of Allelic Homologous Recombination (AHR) within the affected region. However, recombination rate estimates for 22q11.2 region show that neither average recombination rates in the 22q11.2 region or within LCR22-2 (the LCR implicated in most deletions and duplications), are significantly below chromosome 22 averages. Furthermore, LCR22-2, the repeat most frequently implicated in rearrangements, is also the LCR22 with the highest levels of AHR. In addition, we find recombination events in the 22q11.2 region to cluster within families. Within this context, the same chromosome recombines twice in one family; first by AHR and in the next generation by NAHR resulting in an individual affected with the del22q11.2 syndrome.

**Conclusion:**

We show in the context of a first high resolution pedigree map of the 22q11.2 region that NAHR within LCR22 leading to duplications and deletions cannot be explained exclusively under a hypothesis of low AHR rates. In addition, we find that AHR recombination events cluster within families. If normal and aberrant recombination are mechanistically related, the fact that LCR22s undergo frequent AHR and that we find familial differences in recombination rates within the 22q11.2 region would have obvious health-related implications.

## Background

Low copy repeats (LCRs), are 10 to 400 kb long DNA blocks with a complex internal organization that show more than 95% identity between copies. Non-allelic homologous recombination (NAHR) between LCRs has been seen to mediate deletions and duplications in many genomic disorders. All of this supports the view that LCRs are essential players in a common mechanism that causes most genomic syndromes [1]. The 22q11.2 region contains at least 8 LCR22s, four of which are localized within or flanking the most frequent deletions. These LCR22s have been labeled from centromere to telomere: LCR22-2 (or LCR22-A); LCR22-3a (or LCR22-B); LCR22-3b (or LCR22-C); and LCR22-4 (or LCR22-D) [2-4].

**Figure 1:**
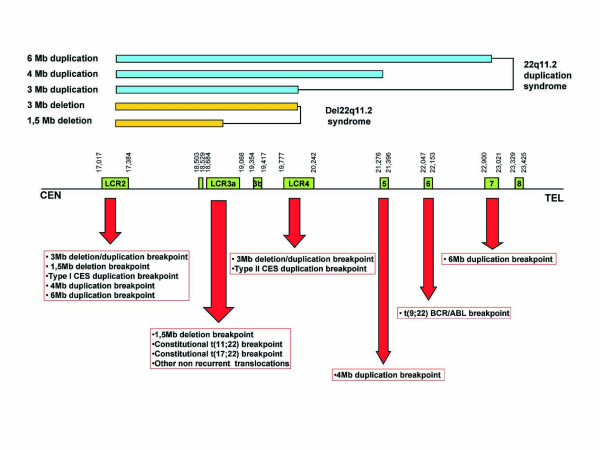
**Map showing recurrent breakpoints of germline and somatic deletions, duplications and translocations that have been described within the 22q11.2 region**. Yellow lines indicate the length of deletions and blue lines the length of duplications (CES: Cat eye syndrome).

The 22q11.2 region undergoes a great amount of germline and somatic rearrangements, and can be classified as one of the most unstable regions of the human genome. As shown in Figure 1, many congenital anomalies are caused by rearrangements within the 22q11.2 region and practically all deletions, duplications and translocations show breakpoints within LCR22s. The del22q11 syndrome is the most frequent of all of these with an estimated *de novo *frequency of 1/4000 live births and it incorporates classical clinical disorders such as DiGeorge syndrome, Conotruncal Anomaly Face syndrome, or Velocardiofacial syndrome (DG, MIM 188400; CTF, MIM 217095; VCF, MIM 192430) [5].

The great majority of patients suffering of the del22q11.2 syndrome (97–98%) have a proximal breakpoint within LCR22-2, whereas the distal breakpoint can vary and in 90% of patients it falls within LCR22-4 producing a 3 Mb deletion (Typical deleted region; TDR); or in 7% of patients within LCR22-3a producing a 1.5 Mb deletion (Figure 1) [3,6,7].

Most deletions in the 22q11.2 region are the result of NAHR between homologous chromosomes (interchomosomal recombination), as opposed to NAHR within the same chromosome (intrachromosomal recombination) [6,8]. Deletion and duplication in interchromosomal NAHR are reciprocal products of the same crossover event and current models predict that they should be equally frequent. In support of this prediction, recent studies have found patients with duplications that are the result of NHAR between the same LCR22s implicated in deletions (Figure 1) [9,10].

Meiotic allelic homologous recombination (AHR) insures proper segregation of chromosomes to gametes and results in the exchange of DNA segments between pairs of homologous chromosomes without loss or gain of genetic material. AHR events are not distributed evenly across the genome and concentrate in hotspots that are 1 to 3 kb in length. In fact, 80% of recombination is calculated to take place in 10–20% of the total genome sequence. However, frequency of recombination within these hotspots can vary by several orders of magnitude, ranging from rates below the genome average (0.3 fold) to frequencies that are far above (120 fold) [11,12].

Not much is known on the relation between AHR and NAHR within the regions implicated in recurrent genome rearrangements. NAHR hotspots that have been studied so far seem to be localized within larger regions that show low rates of AHR during meiosis [13,14]. Because of this observation, it has been proposed that mispairing between non-allelic copies of LCRs may be facilitated by reduced recombination within these regions, leading to unequal crossing over between non-allelic LCRs [1].

There are three available methods that can be used to characterize recombination in the human genome: single-sperm typing, population-based linkage disequilibrium (LD) maps and pedigree-based maps. Single-sperm typing is the method of choice to infer contemporary recombination rates at a very fine scale because it allows typing of large sample sizes. However, single sperm typing detects recombination within short intervals and needs previous information on the location of recombination hotspots and it does not detect female recombination. Population-based LD maps provide an overview of the recombinational history reflecting transmissions from many individuals over thousands of generations and thus reflect only historical recombination rates. These maps although cannot distinguish male and female recombination events, are good predictors of contemporary recombination rates at a scale larger than 5 Mb and detect most (but not all) contemporary hotspots [12,15]. Finally, pedigree-based maps, although inevitably based on relatively few meiosis, have been shown to give rough but reliable estimates of average recombination rates within large regions and to give an overview of the position of recombination hotspots [14,16,17]. Furthermore, a unique feature of pedigree maps is that they allow for the characterization of sex-specific recombination because female and male recombination events can be discriminated.

As a first step towards the characterization of recombination rates and breakpoints within the 22q11.2 region we have constructed a high resolution recombination breakpoint map based on pedigree analysis and a population-based historical recombination map based on LD analysis.

## Methods

### Patients, and samples

Samples from patients and their families were obtained after informed consent. Ethical approval was obtained for this study from the Committee for Ethical Clinical Research of the Government of the Balearic Islands (CEIC). Research was performed in compliance of the Helsinki declaration.

### Position and length of LCR22s

Map construction requires the precise location of low copy repeats within the studied region. Although this had been done before [2,3,18], we determined the precise boundaries of the repeats within the context of the human genome sequence draft (May 2004 release)[19] to be able to locate new markers and recombination breakpoints in reference to each LCR22. For this purpose, 25 kb windows of genomic sequence that had been filtered out of interspersed sequence repeats (between positions 16 and 23 Mb), were compared with the complete human genome sequence draft using the BLAT and BLAST programs. This strategy enabled us to detect identical or near-identical sequences that were located in other genomic positions on chromosome 22 or other chromosomes and hence constituted low copy repeats.

By this approach we identified the boundaries of all the previously described LCR22s, as well as a new previously non-described small low copy repeat. This new LCR22 is approximately 25 kb-long and is positioned between LCR22-2 and LCR22-3A at positions 18.503114-18528713 and is composed by a sequence that is duplicated only within LCR22-6 at positions 22045473-22069076 with a 93% identity. The repeated sequence includes the 3' end of the *ZDHHC8 *gene (exons 8–11) and a predicted gene with an unknown function. Interestingly, the *ZDHHC8 *gene which encodes a putative transmembrane palmitoyltransferase has been proposed to contribute to susceptibility to schizophrenia associated with the 22q11.2 region [20]. The other repeats were found to have the following positions and sizes: LCR2 17016923-17384345 (367, 42 KB); LCR3A 18684169-19068000 (383,381 KB) (this an estimation because of a gap in the sequence); LCR3B 19353714-19416000 (62,286 KB); LCR4A 19777000-20121932 (344,932 KB); LCR4B 20137031-20241670 (104,639 KB); LCR5 21276000-21396500 (120,5 KB) and LCR6 22047000-22153000 (106 KB).

### New marker design

New polymorphic simple sequence repeats (CATCH markers) were identified using the Tandem Repeats Finder and the RepeatMasker tracks of the May 2004 release of the human reference sequence available at the University of Santa Cruz (UCSC) Genome Browser [19]. Once a repeat was identified in the region of interest, the design of primers for PCR was performed using the Oligos 9.6 program [21]. Interspersed repetitive sequences identified by RepeatMasker were avoided when possible as primer annealing sequences. Finally, sequences flanking the repeat were checked against the human genome using the BLAST and BLAT programs [19,22] to ensure it was a single copy marker. PCR conditions and annealing temperatures were determined empirically based on program predictions (see Additional File 1).

### Pedigree-based linkage map construction

Fourteen families and a total of 152 genomic DNAs of family members were typed to detect recombination events within the 22q11.2 region. Families were Spanish from Mediterranean regions (Balearic Islands and Catalonia). Ten families included 3 generations or more, and the other four included both parents and at least three offspring. Two of these families had a member affected with the del22q11.2 syndrome, while the other families had no known disease associated to the 22q11.2 region. In total, 204 informative meiosis (83 female, 98 male, and 23 of unknown sex) were typed with 62 different microsatellite polymorphic markers (see Additional file 1) over a region spanning 6,5 Mb. Deleted chromosomes were not counted as informative meiosis because they were the product of an illegitimate recombination (NAHR) event. DNAs were initially typed with 6 microsatellite markers (*D22S420*, *D22S427*, *D22S264*, *D22S308*, *D22S303 *and *D22S1174*) to determine the most likely haplotypes, detect recombination events within the 22q11.2 region, and avoid non-detection of double crossovers. When outlying markers *D22S420 *or *D22S1174 *were not informative they were replaced by the nearest informative marker available. In consequence, the total number of informative meiosis is 204 between markers *D22S427 *and *D22S425*, but this number decreases towards the outflanking regions. Recombination breakpoints were then positioned by typing all available markers within the interval where the breakpoint had been initially localized.

Haplotypes were determined with the help of the grandparental genotypes, when available, and by minimizing the number of double crossovers. In three of the families, the first generation consists of three siblings or more and haplotypes can be assigned unambiguously, although it can not be determined if it was a female or a male recombination event. Genetic distances between markers were calculated by dividing number of recombination events within the interval by the total number of informative meiosis for the same interval.

We pooled our data with that of the CEPH website [23] which includes data for markers flanking but not within the TDR for the following families: 17, 66, 102, 884, 1331, 1332, 1333, 1334, 1340, 1341,1346, 1347, 1358, 1362, 1408, 1413, 1416, 1420,1423, 1454 (236 meiosis). In total, within these CEPH families, we identified 33 recombination events, that because of the paucity of markers, we were not able to narrow down recombination breakpoints to small sized segments. As an exception, 3 breakpoints were localized to LCR22-2 (2 male and 1 female) and 1 breakpoint to LCR22-4 (male). All this data was pooled together with that of our linkage map to calculate recombination rates within the 22q11.2 region and within each of the LCR22s.

### Population-based linkage-disequilibrium SNP map and hotspot estimation

Population recombination parameters were determined from data available at the public HapMap Project (public data release #21 October 2004)[24]. We selected a total of 2074 SNPs between positions 16198487 to 23198486. Genotypes were recorded for 60 unrelated individuals corresponding to parents of the CEPH dataset (northern and western European ancestry).

Estimation of recombination parameters was done using the PHASE v2.1.1 software which calculates linkage disequilibrium (LD) patterns among SNP pairs and derives two parameters for each interval: the population recombination rate (ρ) which reflects the demographic history of the population and a measure of how recombination varies within the same interval (λ)[25,26]. To this end we divided the 22q11.2 region into 15 400-kb non overlapping windows, except for those containing LCR22-2, LCR22-3A and LCR22-4 that were of 800-kb. An additional window of 437 kb located within the *IGL locus *region was considered.

Two models of variation of recombination were considered, the general model which is the default model for PHASE v2.1.1 (option -MR0) and the simple hotspot model (option -MR1). The first model considers that the recombination rate in a genomic region is a function of parameters ρ and λ. The second model considers that recombination rate in a genomic region equals ρ, except in the hotspot region in which recombination rate equals λρ. We used the default priors for recombination parameters defined in the PHASE v2.1.1 software [25]. For each input file, we ran PHASE five times and selected the data from the run with the best average value for the goodness of fit (option -x5). The final run for the whole dataset was run 10 times longer to obtain better estimates of the recombination parameters (option -X10). Data was analysed from the recombination output file from which we computed the median of the 1000 draws from the posterior distributions of ρ and λ. We computed ρ at each SNP interval by the product of λρ. Under the simple hotspot model, we estimated the posterior probability of both, λ greater than 10 and λ greater than 100 and computed the associated Bayes Factor (BF). BF measures the strength of evidence for a hot spot by computing the probability of obtaining the data if a hot spot is present divided by the probability of obtaining the data if a the hot spot is not present [25]. We considered intervals of the hotspot to extend from the 5th percentile of the posterior probability for the left end to the 95th percentile of the posterior probability for the right end.

### Statistical calculations

The expected number of families having 0, 1, 2 and ≥ 3 recombination events in the TDR region was modeled following a Poisson distribution, assuming a random occurrence of a recombination event in a family. As the opportunity to observe a recombination event in a family depends on the number of meiosis observed, we only consider those families from witch we have observed a minimum of 10 meiosis. From the 18 families that fulfill this criterion, we estimated a frequency of 1.5 recombination events per family (18 families, with a mean number of 20 meiosis per family and a total of 27 recombination events). The observed number of families having 0, 1, 2, and ≥ 3 recombination events was compared with their expected values assuming a Poisson distribution with a chi square test with 2 degrees of freedom. We also tested for differences among observed and expected values by the mean distance test of Poissonity proposed by Szekely and Rizzo [2004]. This test is a goodness-of-fit test based on the estimator of the cumulative distribution function. The test statistic is a Cramer-von Mises type of distance and it is implemented in the R Software by parametric bootstrap. The poisson.mtest of R was run with 10000 replicates.

## Results

### Breakpoint distribution

**Figure 2:**
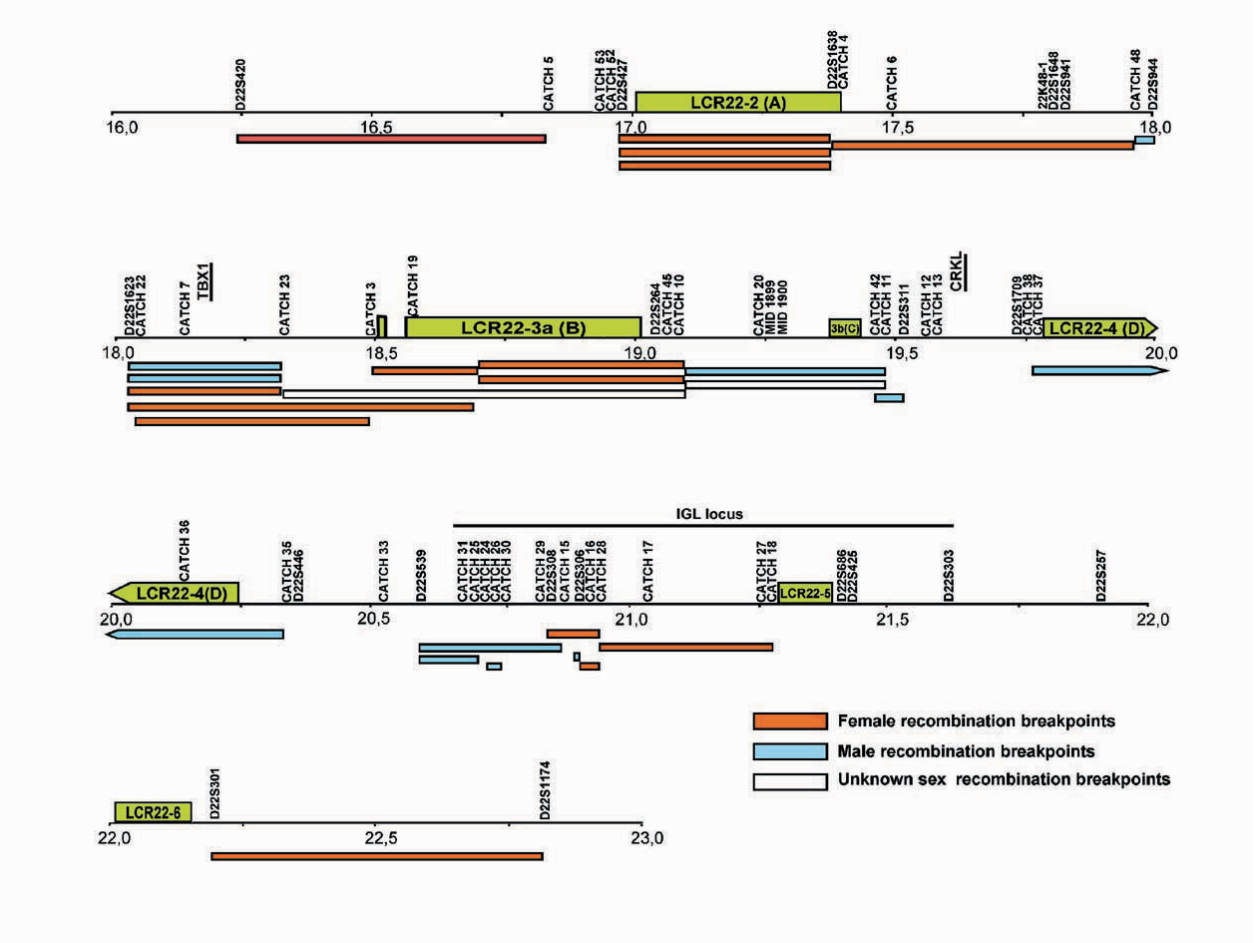
**Pedigree-based recombination breakpoint map based on the typing of 62 polymorphic markers (38 designed for this study; CATCH markers) in 14 extensive families totaling 204 meiosis**. We localized a total of 27 single recombination events. The 22q11.2 region is drawn to scale including the locations of the LCR22s (green boxes) and the position of the polymorphic markers used. Blue, red and white lines represent regions to where male (blue), female (red) and unknown sex (white) recombination breakpoints have been narrowed down. Genes *TBX1 *and *CRKL *that are implicated in the clinical manifestations of the del22q11.2 syndrome are shown, as is the *IGL *locus also mentioned in the text.

The pedigree map is based on 204 informative meiosis that were typed with 62 different microsatellite polymorphic markers (38 designed for this study; CATCH markers) (see Additional File 1) over a region spanning 6.58 Mb (Figure 2). This approach led to the detection of 27 single recombination events and no double crossovers. Markers are not distributed uniformly over the entire 22q11.2 region. Regions with a high marker density, as for example the Immunoglobulin lambda locus (*IGL*), have permitted the location of 5 breakpoints to segments of 54 kb or less (8.6 kb the smallest) (Table 1). Overall, the average marker density between *D22S427 *and *D22S1174 *is approximately of 1 marker every 85 kb if we exclude the genomic sequence that corresponds to the repeated DNA of the LCR22s and the median size of breakpoint intervals is of 338 kb.

**Table 1:** Genomic features of small regions containing breakpoints in family data

Family data				Hotspot prediction from HapMap data (Phase 2.1, MR1 model)					Hotspot prediction at UCSC^a^	
		
Outlying markers (centromeric, telomeric)	Position (bp)	Size crossover region	Crossovers detected	Hotspot boundaries	Hotspot with (bp)	Hotspot intensity (λ, 95%CI)	Bayes Factor (log)		Hotspot predicted	Recombination intensity (cM/Mb)
									
							λ > 10	λ > 100		
		
CATCH 48 D22S944	17,95198 17,98496	32,98	1	17,963500 17,971900	8400	762 (466:990)	3.00	3.00	Yes	17.36 (Hapmap) 7.02 (Perlegen)
CATCH 42 D22S311	19,449222 19,503575	54,353	1	19,229800 19,237700	7900	2.6 (1:8)	-1.45	-3.00	No	3.02 (Hapmap) 0.54 (Perlegen)
CATCH 26 CATCH 30	20,706255 20,740881	34,626	1	20,634600 20,640300	5700	8.5 (1.1:32)	-0.54	-2.70	Yes	40.53 (Hapmap) 17.61 (Perlegen)
D22S306 CATCH 16	20,887523 20,896124	8,601	1	20,881700 20,890000	8300	131 (17:307)	3.00	0.09	Yes	10.62 (Hapmap) 46.50 (Perlegen)
CATCH 16 CATCH 28	20,896124 20,929869	33,745	1	--	--	--	--	--	Yes	9.38 (Hapmap) 15.67 (Perlegen)

^a ^based on hotspot information and SNP-based recombination rates available in the UCSC genome browser.

Recombination events do not spread evenly within the studied region and there are segments where crossovers tend to cluster and segments with little or no recombination. Furthermore, we find that we can divide the 22q11.2 region into segments that alternatively undergo male or female recombination with very little overlap (Figure 2). The regions that go from position 16.23 to 17.95 Mb (*D22S420*-*CATCH48*), 18.31 to 19.09 Mb (*CATCH23*-D22S264) and 20.88 to 22.81 Mb (*D22S306*-*D22S1174*) are female-specific with no male recombination. Segments between 19.09 and 19.5 (*D22S264*-*D22S311*); 19.74 and 20.34 (*CATCH38*-*CATCH35*), and 20.58 and 20.81 (*D22S539*-CATCH29) are sections of exclusively male recombination. There are only two regions that present both male and female recombination events, those between 18.02 and 18.31 (*D22S1623*-*CATCH23*) and 20.81 and 20.89 (*CATCH29*-*CATCH16*).

### AHR rates in the 22q11.2 region

Average recombination rates in the 22q11.2 region were calculated by pooling our data with that available for the CEPH families at the CEPH Genotype database browser V2.1. In this way, we improved the precision of our estimates by increasing the sample size to 440 meioses and 60 recombination events. Based on these data the sex-averaged recombination rate for the analyzed region (6. 58 Mb, positions 16233835-2281304) is of 2.1 cM/Mb (95% confidence intervals (CI): 1.6–2.76). If we concentrate on the 3 Mb TDR, the sex-averaged recombination rate increases to 2.6 cM/Mb (CI: 1.8–4.4). On the other hand, sex-specific female and male rates in the 6.5 Mb 22q11.2 region are 2.9 cM/Mb (CI: 1.9–4.5) and 1.4 cM/Mb (CI: 0.7–2.5), respectively. In the TDR sex-specific rates increase to 4.0 cM/Mb (CI: 2.2–6.9) in females and 2.0 cM/Mb (CI: 0.9–4.2), in males. Neither 22q11.2 recombination rates, nor those of the 3 Mb TDR included in the larger 6, 58 Mb region, differ significantly from chromosome 22 averages described in previous studies [27,28]. From this we can conclude, that overall AHR within the 22q11.2 region or within the TDR are not below chromosome 22 averages.

**Figure 3:**
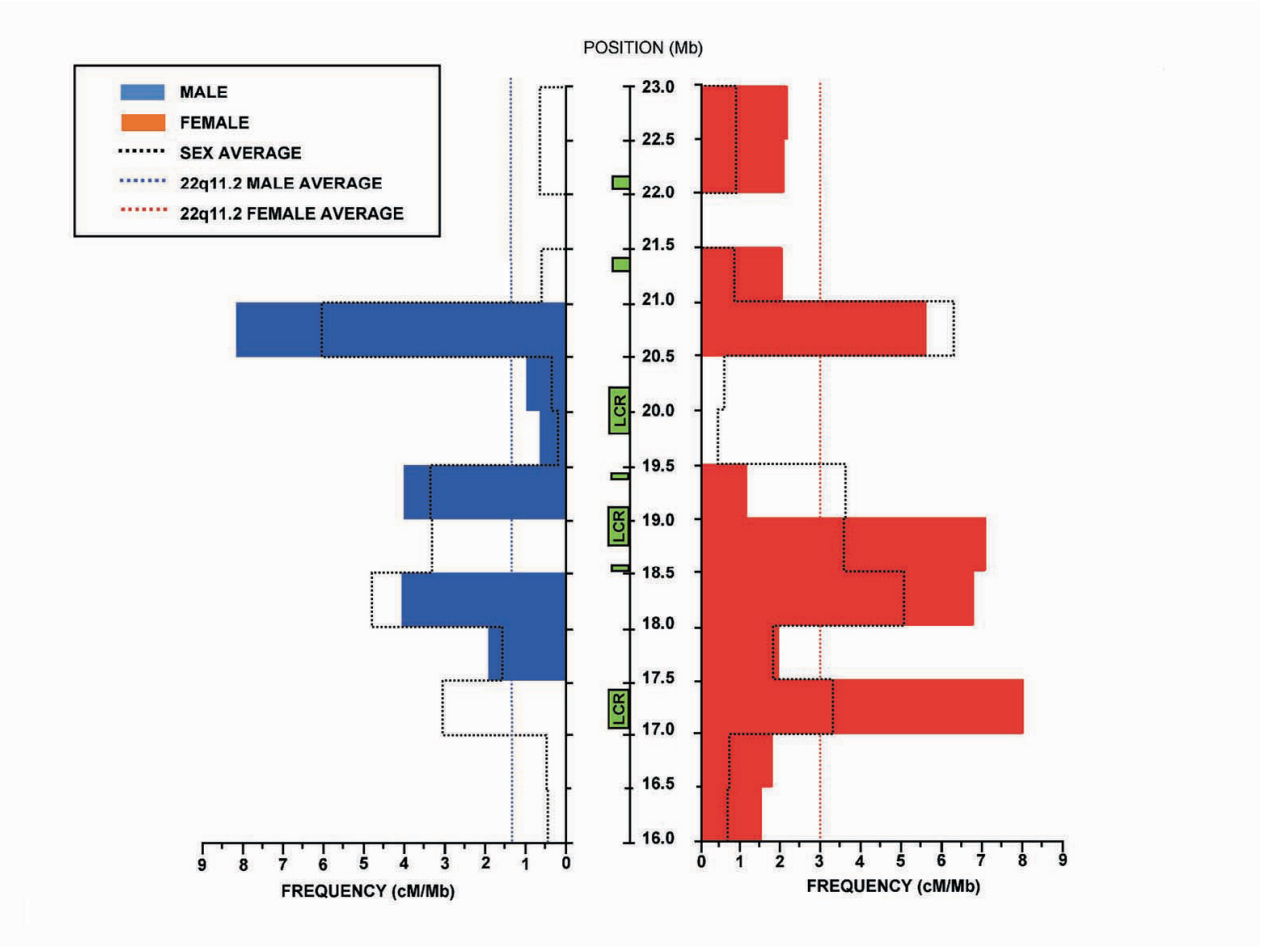
**Variation of recombination rates along the 22q11.2 region**. We show 14 intervals of 500 kb and calculated the frequency of recombination (cM/Mb) for each. On the left, in blue we show male recombination rates and on the right, in red, female recombination rates. Dotted lines show sex-averaged recombination, while dotted red and blue lines show the female and male recombination averages for the entire 22q11.2 region.

Our pedigree map allows the location of recombination breakpoints with a high resolution but sample size does not permit the accurate measure of recombination rates at a fine scale. However, recombination rate inaccuracies can be reduced by decreasing the resolution and for this reason we analyzed recombination rates in 14 intervals of 500 kb each [16]. This analysis provides an estimation of the variation of contemporary recombination rates within the 22q11.2 region (Figure 3). We find 3 regions to display high recombination rates. The first of these regions has an estimated recombination frequency of 3.2 cM/Mb (CI: 1.1–8.4), extends from position 17 to17.5 Mb and includes LCR22-2. The second spans from position 18 Mb to 19.5 Mb and shows a peak at position 18–18.5 with a rate of 4.9 cM/Mb (CI: 2.2–11.4). This region includes LCR22-3A and B. The third region between positions 20.5 and 21 Mb shows the highest calculated recombination rate of 6.1 cM/Mb (CI: 2.8–12.7), and includes part of the immunoglobulin light chain (IGL) locus.

### Coincidence between breakpoints and historical recombination hotspots

**Figure 4:**
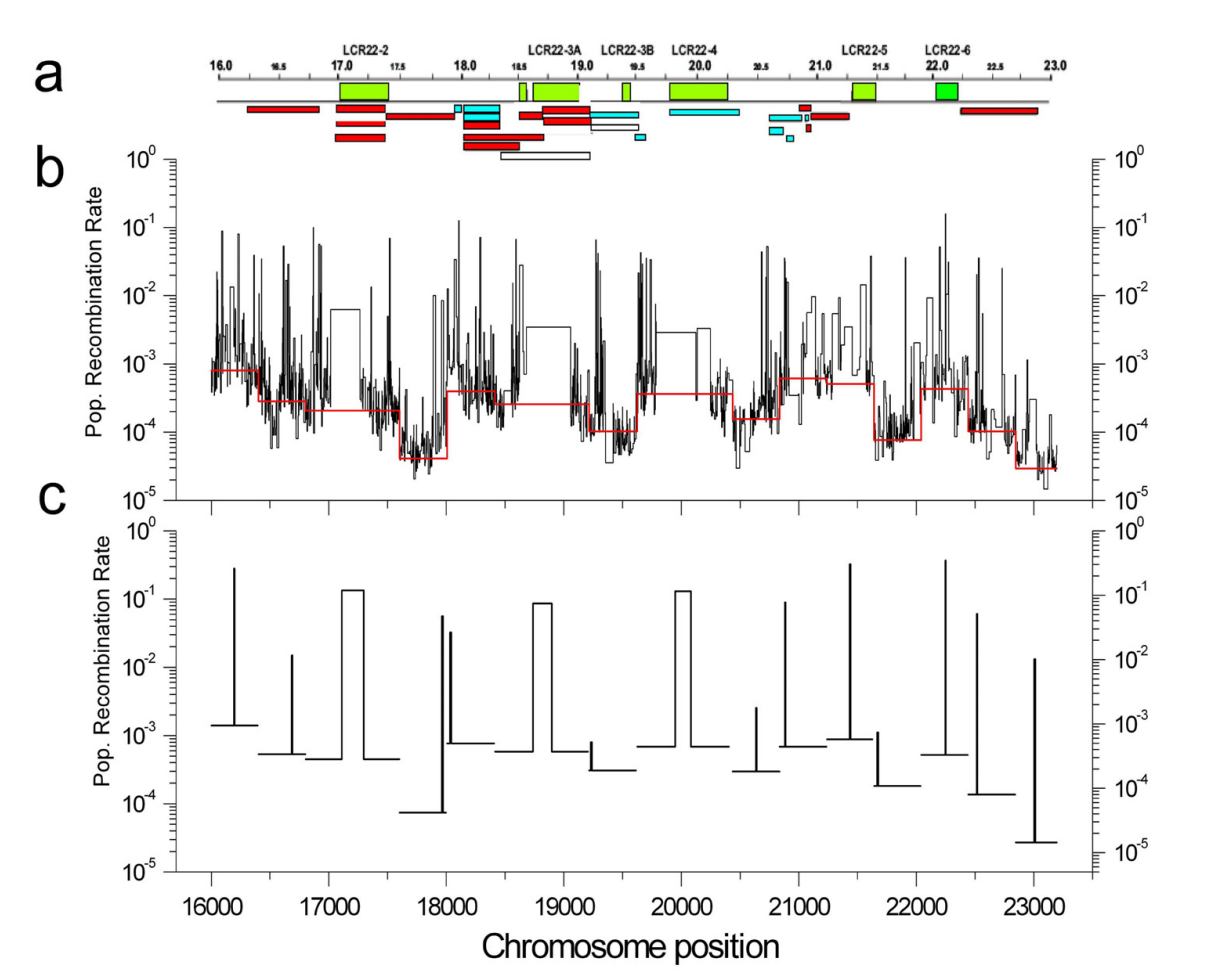
**Comparison between the population-based linkage-disequilibrium map and the pedigree-based linkage map**. **A**, An outline of the pedigree-based linkage map according to Figure 1. **B**, Population based linkage-disequilibrium SNP map. The map was constructed using genotype information publicly available from the HapMap project site , for a total of 2074 SNPs of 60 unrelated individuals corresponding to parents of the CEPH dataset (Utah residents with ancestry from northern and western Europe; CEU). Background population recombination rate (ρ) per base pair and the factor by which the recombination rate between two adjacent SNPs exceeds the background rate (λ) were estimated for each window by the PHASE v2.1.1 software using the general model for recombination rate variation (-MR0 option). We plotted the ρ value for each window (red line) and the λρ value for each SNP interval (black line). **C**, The presence of a recombination hotspot was estimated for each window using the simple hotspot model for recombination (-MR1 option). We estimated ρ and λ inside the hot spot region and the hot spot boundaries defined by their left and right ends and plotted the λρ value across each window (black lines).

**Figure 5:**
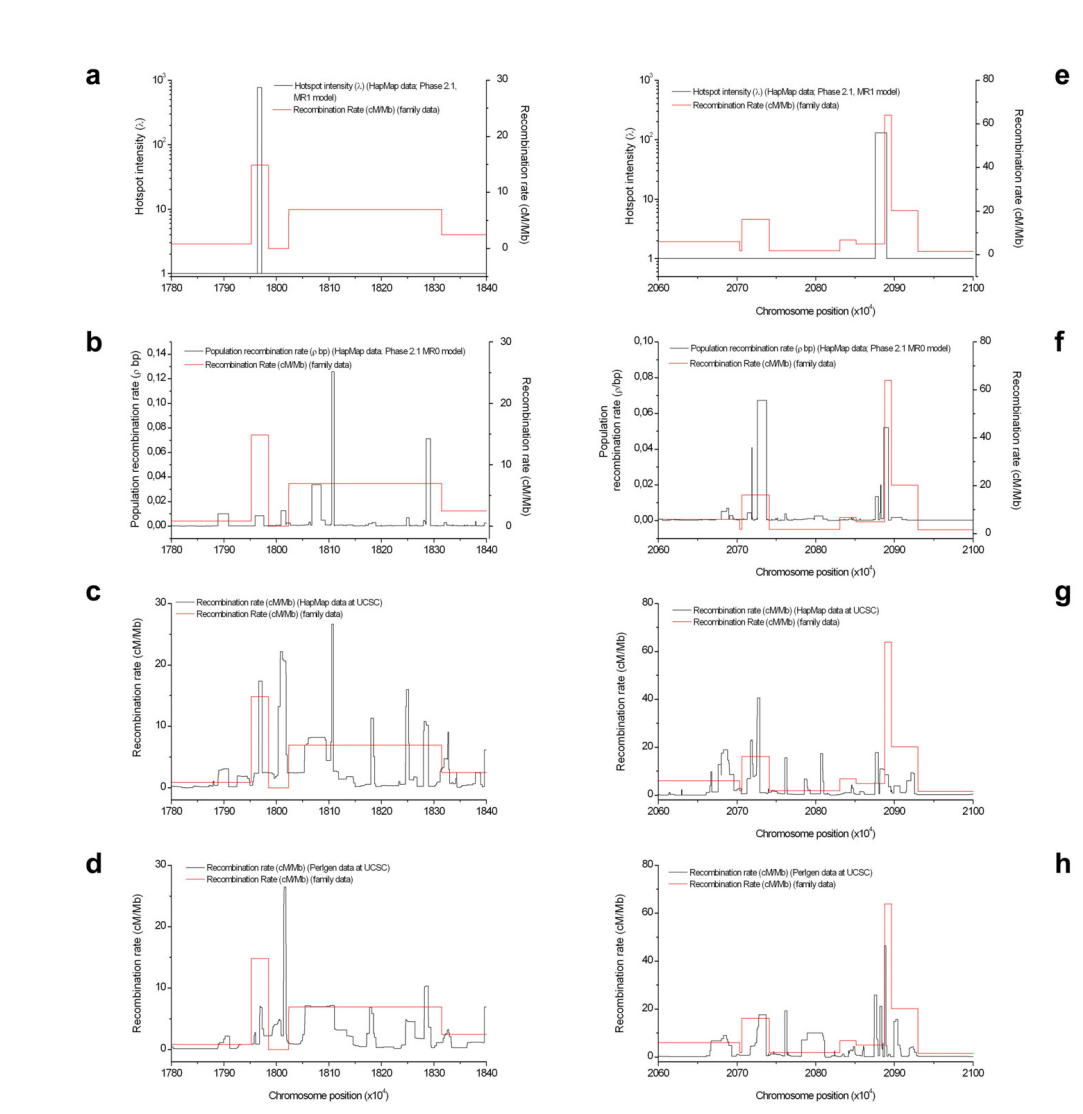
**Hotspot prediction and comparison between historical and contemporary recombination rates in two regions of high marker density in the pedigree-based map**. Charts **a-d **are for the region between positions 17,80 and 18,40 Mb and **e-h **for the region between positions 20,60 and 21,00 Mb. In all charts recombination rates based on family data are shown in red. Charts **a **and **e **shows the prediction of hotspot intensity and location (PHASE 2.1.1 software, -MR1 option) in the region. Charts **b **and **f **show population recombination rate profile (PHASE 2.1.1 software, -MR0 option) based on our analysis. Charts **c **and **g **show population recombination rate profiles as shown in the UCSC genome web tracks based on Hapmap data. Charts **d **and **h **show population recombination rate profiles as shown in the UCSC genome web tracks based on Perlegen data.

An LD map was constructed based on 2074 SNPs from the HapMap project (3,5 kb resolution) that were analyzed in 400–800 kb windows using the general model for recombination rate variation of the PHASE 2.1.1 software (Table 2 and Figure 4) [25,26]. Regions with a high density of markers in the pedigree map were used to determine if contemporary breakpoints coincide with peaks of historical recombination. The analysis of two of such regions (17, 80 to 18, 40 and 20,60 to 21,00 Mb) found that small breakpoint intervals in the pedigree map coincide in all cases with peaks of historical recombination (Figure 5). Furthermore, small breakpoint segments can be used to interrogate if recombination breakpoints that were found in the pedigree map coincide with historical recombination hotspots and thus potentially reflect the activity of contemporary hotspots. When such analysis was performed, we found that 4 out of 5 breakpoint segments smaller than 54 kb coincide with historical hotspots predicted by our LD map and those at the UCSC genome browser based both on HapMap and Perlegen data (Table 1 and Figure 5) [29].

**Table 2:** Descriptive data used for the analysis of the Hapmap population-based recombination profile of the 22q11.2 region

**Characteristics of windows**							**General model for recombination (MR0)^(1)^**	**Simple hotspot model for recombination (MR1)^(2)^**						
	**Chromosome position**								**Hotspot boundaries^(4) ^**(chromosome position)				**Bayes factor (BF)^(6) ^**(log BF)	
**Window**	begin	end	**Window length **(bp)	**SNPs/window**	**SNP density **(bp/SNP)	***LCR containing windows***	**Population recombination rate (ρ/bp^(3)^**	**Population recombination rate (ρ/bp^(3)^**	***left***	***rigth***	**Hotspot width **(bp)	**Hotspot Intensity^(5) ^**(λ)	**λ > 10**	**λ > 100**

I	16000790	16396314	395524	126	3139		0.00079688	0.00139749	16192800	16198100	5300	200(70:438)	3.00	0.59
II	16402210	16801972	399762	187	2138		0.00028288	0.00053267	16683800	16691200	7400	28.2(1.2:145)	-0.03	-1.13
III	16802997	17601177	798180	275	2902	LCR22-2	0.00020637	0.00044908	17111470	17296800	185330 (***LCR22-2***)	298(66:955)	3.00	1.05
IV	17603290	17998020	394730	137	2881		0.00004115	0.00007356	17963500	17971900	8400	762(466:990)	3.00	3.00
V	18006919	18406881	399962	157	2548		0.00039653	0.00076869	18032000	18041400	9400	42(1.8:145)	0.21	-1.00
VI	18414421	19205939	791518	173	4575	LCR22-3A	0.00025670	0.00058289	18737180	18897600	160420 (***LCR22-3A***)	147(12:805)	1.67	-0.38
VII	19215813	19613455	397642	141	2820	LCR22-3B	0.00010245	0.00030662	19229800	19237700	7900	2,6(1:8)	1.45	-3.00
VIII	19624609	20406127	781518	158	4946	LCR22-4	0.00036461	0.00068812	19947500	20081300	133800 (***LCR22-4***)	188(10:903)	1.26	-0.43
IX	20435862	20835601	399739	129	3099		0.00015527	0.00029812	20634600	20640300	5700	8,5(1,15:32,5)	-0.54	-2.70
X	20835924	21235614	399690	81	4934		0.00061587	0.00068451	20881700	20885700	4000	131(417:307)	3.00	0.09
XI	21238221	21625884	387663	68	5701	LCR22-5	0.00051035	0.00087814	21432500	21439900	7400	370(9:890)	1.24	0.39
XII	21640460	22037074	396614	139	2853		0.00007681	0.00018216	21668195	21676200	8005	6(1.1:19)	-0.78	-3.00
XIII	22040766	22438098	397332	97	4096		0.00042922	0.00052211	22247500	22250300	2800	702(391:966)	3.00	3.00
XIV	22441140	22840845	399705	124	3223	LCR22-6	0.00010236	0.00013618	22514100	22519600	5500	447(211:780)	3.00	2.22
XV	22844929	23194681	349752	82	4265		0.00002937	0.00002733	23002500	23014000	11500	480(130:898)	3.00	1.63
**Total region**	16000790	23194681	7193891	2074	3469		0.000290	0.000516						

(1) Background population recombination rate (ρ) was estimated for each window using genotype data available at the HAPMAP project for CEU unrelated individuals, genotypes were analyzed by the PHASE v2.1.1 software using the general model for recombination rate variation (-MR0 option) after performing 1.000 iterations to estimate posterior distribution of recombination parameters (-X10 option). All the analyses were performed running PHASE five times and selecting the best average value for the "goodness of fit" (-x5 option). We computed all the recombination parameters estimates based on the recombination output file, which provides a sample from the posterior distribution of the background recombination rate (ρ) per base pair and the factor by which the recombination rate between two adjacent SNPs exceeds the background rate (λ).

(2) The presence of a recombination spot was estimated for each window by the PHASE v2.1.1 software using the simple hotspot model for recombination (-MR1 option) after performing 1000 iterations to estimate posterior distribution of recombination parameters (-X10 option), We computed all the recombination parameters estimates based on the hotspot output file, which provides a sample from the posterior distribution of the background recombination rate (ρ) per base pair, the factor by which the recombination rate in the hotspot exceeds the background rate (λ) and the left and right ends of the hotspot.

(3) From the estimated recombination parameters we computed the median ρ of each window and the median ρ of the 22q11.2 region by a weighted average of the median ρ of each window.

(4) Hotspot boundaries were considered to extend from the 5th percentile of the posterior probability for the left end to the 95th percentile for the right end.

(5) Of the 1.000 draws from the posterior distribution of λ we computed the median and the 5th and 95th percentile.

(6) From the posterior distribution of λ, we computed the probability of both: λ > 10 and λ > 100 and computed the associated Bayes Factor (BF) to asses the statistical support of a recombination spot. A BF > 10 (Log BF > 1) was taken to indicate the presence of a recombination spot, that was considered as a warm spot if BF > 10 (Log BF > 1) computed under P(λ > 10) and hotspot if BF > 10 (Log BF > 1) computed under P(λ > 100).

### Concordance between AHR and NAHR

LCR22s are frequent sites of illegitimate recombination that cause deletions and duplications [3-6,9]. To determine if LCR22s are also sites of frequent AHR meiotic recombination, we made primer sets that amplify polymorphic markers flanking the different LCR22s (Additional file 1 and Figure 2). Pedigree recombination analysis shows that LCR22-2 displays the highest recombination rates of all the LCR22s: 4, 54 cM/Mb (CI: 2, 35-9, 7 cM/Mb); LCR22-3a, shows a recombination rate of 2,76 cM/Mb (CI: 1, 35-5, 2 cM/Mb) and LCR22-4 of 1,91 cM/Mb (CI: 1, 15-6, 0 cM/Mb). No recombination events were localized within LCR22-5 or LCR22-6.

To assess the support in the HapMap LD map we constructed for each LCR22 being "hot" regions for AHR, we used the PHASE 2.1.1 program (option -MR1) to estimate the most probable hotspot within each of the 400–800 kb windows into which the 22q11.2 region was divided. The program predicts LCR22-2, LCR22-3A and LCR22-4 to contain the main recombination hotspot of their 800 kb window (Table 2), but not LCR22-5 and LCR22-6. LCR22-2 is again the one with the highest recombination intensity (λ = 298; 298 times the background recombination of the window that contains LCR22-2) and likelihood (logBF = 3).

### Family clustering of NAHR and AHR events

**Figure 6:**
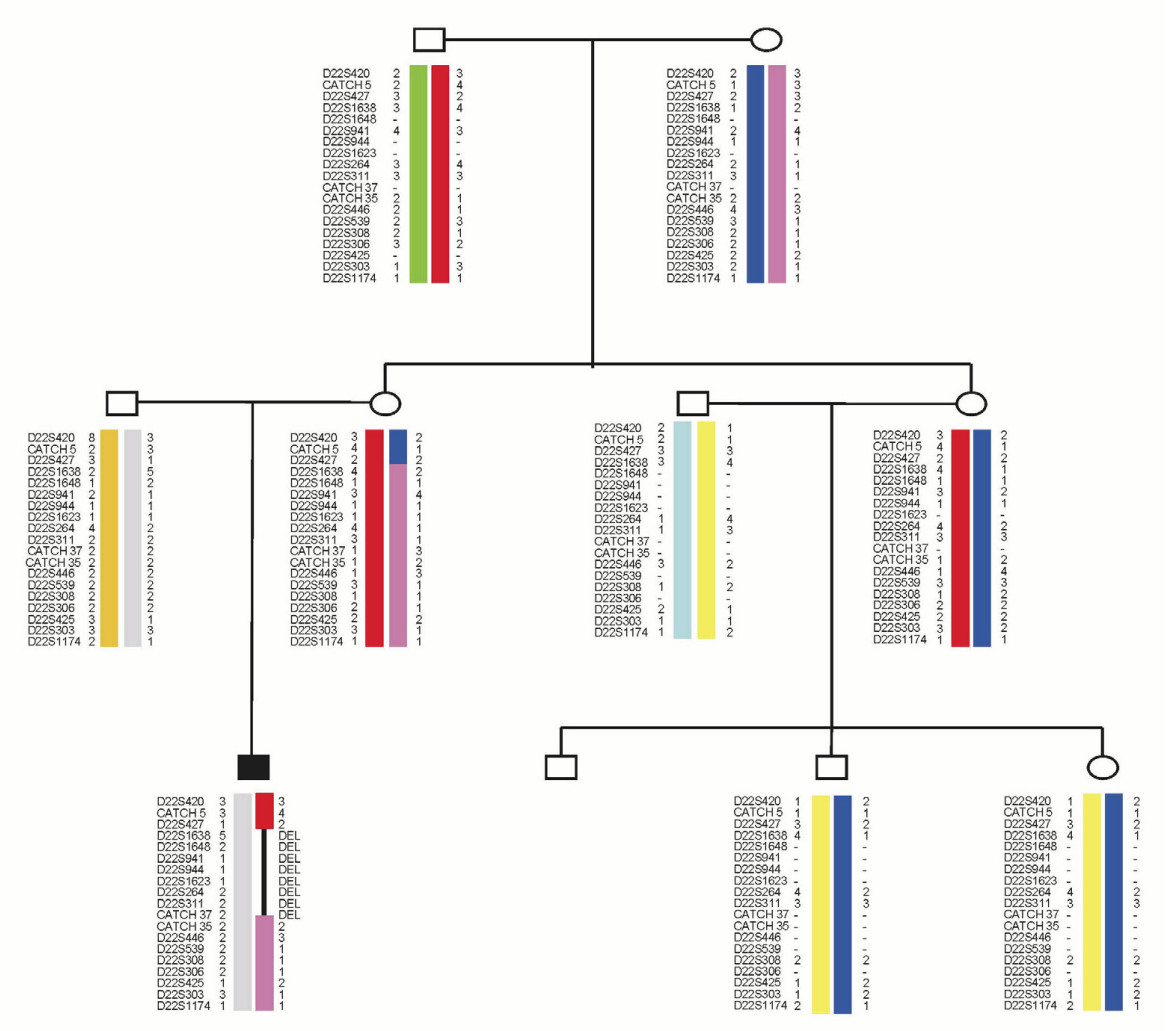
**Pedigree and haplotype data of a family with a member affected with the del22q11.2 syndrome and carrying the 3 Mb deletion caused by an interchromosomal NAHR event**. The NAHR event was of maternal origin and in the previous generation there is a female AHR event within LCR22-2 that did not cause a deletion. The AHR event has been arbitrarily assigned to the mother of the deleted child.

We analyzed recombination events within several three-generation families with a member carrying a 3 Mb deletion caused by interchromosomal NAHR between LCR22-2 and LCR22-4. In one of these families, we found a grand-maternal AHR event within LCR22-2 that had no pathological consequences. In the next generation, the same chromosome involved in this AHR event participated in a NAHR event causing a 3 Mb deletion (pink haplotype in Figure. 6). Moreover, both events were the product of female recombination.

**Figure 7:**
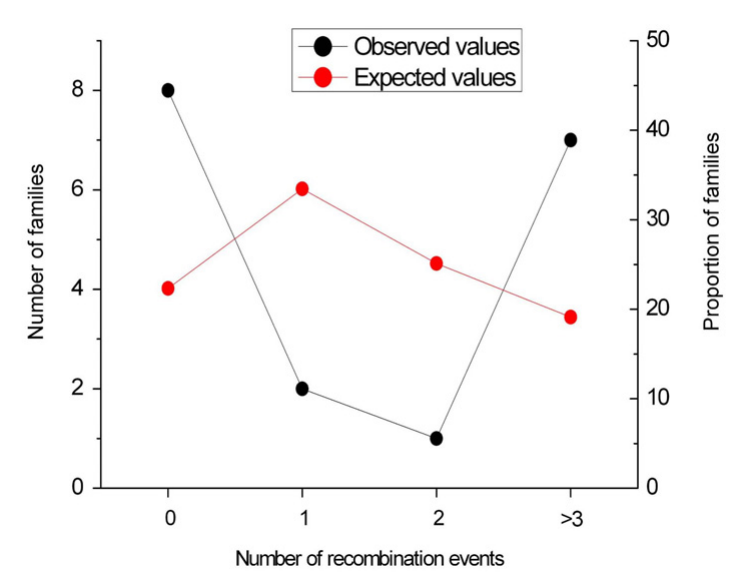
**Recombination events per family within the Typically Deleted Region (TDR) in a sample of 18 large families with 10 or more meiosis analyzed**. Observed values are statistically different from those expected under a Poisson distribution (χ^2 ^= 13.06, 2 df; P = 0.0016 and Mean distance test of Poissonity, P = 0.0033). We take this to indicate that some families have a higher tendency to recombine within the TDR region than others.

This observation suggests that certain haplotypes may be more prone to undergo AHR and NAHR events within the 22q11.2 region. To test this we analyzed the presence or absence of recombination events within TDR in families used to construct the pedigree map. This analysis shows a non random distribution of recombination events per family. Families tend to cluster in two extreme groups: one with no recombination events and another with more than 3 recombination events in this region. To test for the statistical significance of this observation we modeled the expected number of families having 0, 1, 2 and more that 3 recombination events as a Poisson distribution of mean 1.5. In a sample of 18 families, the observed number of families having 0, 1, 2 and more than 3 recombination events was of 8, 2, 1 and 7, respectively (Figure 7). These numbers were significantly different from those expected under a Poisson distribution (4, 6, 4.5 and 2.3, respectively) (χ^2 ^= 13.06, 2 df; P = 0.0016 and Mean distance test of Poissonity, P = 0.0033). This result shows that certain families have a higher tendency to recombine within the TDR region than others.

To explore if there is a genetic basis within the TDR for the observed differences between families, we analyzed microsatellite markers that are located within the TDR in individuals recombining within this region. However, allelic frequencies were not significantly different in these individuals from those of the overall population (data not shown). This indicates that high and low recombining families do not show different origins for the TDR. Therefore, although we have found indications that certain individuals may undergo AHR in the TDR more frequently than others, we have not found any apparent differences within the TDR itself between such individuals.

## Discussion

The knowledge of the detailed contemporary recombinational traits of a genomic region is based on the localization of the exact position of crossovers and the accurate characterization of recombination frequencies. However, previously published genetic maps of chromosome 22 provided very little information on the 22q11.2 region [27,28]. These maps had been obtained within the context of the construction of genome-wide genetic maps and the marker density in the 22q11.2 region was low. In fact, none of them included markers located within the 3 Mb region most frequently implicated in the 22q11.2 deletion and duplication syndrome (typically deleted region; TDR) (Figure 1). In addition, several high resolution maps based on LD data reflecting historical recombination rates have been published [12,25,29,30]. However, correspondence between historical and contemporary recombination rates is not perfect which justifies the need for a recombination map based on breakpoint analysis.

The resolution of a genetic map determines how precisely we know the position at which a crossover is located and is limited by the density of polymorphisms and the distance between the closest markers. On the other hand, the accuracy of the recombination rate of a particular genomic segment is a function of the number of meiotic events studied [16]. The pedigree-based map we present here, although of limited accuracy (204 meiosis), has a much higher marker density (1 marker per 85 kb) than other previous genetic maps of the region [28]. Because of its high resolution our map allows the localization of crossovers to small segments and obtains a comprehensive overview of regions of high and low recombination and of potential recombination hotspots. As a result, we have identified three regions that display higher recombination rates than the others within 22q11.2: one comprising LCR22-2, a second LCR22-3A and B, and a third the IGL locus. In addition, we have seen that male and female recombination is very compartmentalized, with regions containing only male or female crossovers with practically no overlap.

However, the particular traits of the 22q11.2 region warrant a point of caution on recombination rates because there may be inaccuracies in the LCR22 sizes calculated from the available human genome sequence drafts (UCSC; May 2004 release): The size of LCR22-3a may not be exact, as it is an estimation because of a sequence gap. In addition, recently it has been shown that LCR22-2 and LCR22-4 can be polymorphic in size in the normal population (copy number variations; CNV) and thus certain individuals may have larger or smaller LCR22s [31].

Population-based LD maps which model historical recombination rates have been shown to be able to predict most contemporary recombination hotspots [17,25,32]. To determine with increased accuracy and resolution the recombinational traits of the 22q11.2 region we have also constructed a population-based LD map from Hapmap SNP-genotype data (2074 SNPs). This LD map was used to provide support to predictions made by the pedigree map. We find coincidence between small breakpoint segments in the pedigree map and historical recombination hotspots (Table 1 and Figure 5).

Interchromosomal non-allelic homologous recombination (NAHR) between LCR22s has been seen to mediate most of the germline deletions and duplications in the 22q11.2 region. However, not much is known on a possible relation between normal meiotic recombination (AHR) and ectopic recombination (NAHR) in regions implicated in recurrent genome rearrangements. We find some support for a relation between high rates of AHR and NAHR in the 22q11.2 region. Our maps show that LCR22-2, LCR22-3a and LCR22-4 undergo frequent AHR and that LCR22-5 and LCR22-6 support little or no recombination. Interestingly, NAHR between the three LCR22s displaying frequent AHR account for nearly all described deletions in the 22q11.2 region. It is also suggestive that LCR22-2, the LCR that mediates most deletions and duplications within the 22q11.2 region, is also the LCR in which we have found the highest AHR rates. Furthermore, AHR breakpoints detected within LCR22-2 are mostly female and we show through a meta-analysis of published data that there is a significant excess of maternal deletions in del22q11.2 syndrome patients (56%; X^2^: p = 0, 0238) (Table 3). This result indicates that there is also a female bias in NAHR causing de novo 22q11.2 deletions.

**Table 3:** Meta-analysis of the parental origin of *de novo *22q11.2 deletions

Reference	Maternal origin	Paternal Origin
DriscolL et al. 1992 [35]	4	1
Seaver et al. 1994 [36]	4	1
Morrow et al. 1995 [37]	8	7
Demczuk et al. 1995 [38]	16	5
Ryan et al. 1997 [39]	13	24
Bonnet et al. 1997 [40]	11	4
Fokstuen et al. 1998 [41]	5	4
Matsuoka et al. 1998 [42]	28	20
Rauch et al. 1998 [43]	5	3
Baumer et al. 1998 [8]	4	6
Edelman et al. 1999 [3]	2	0
Lu et al. 2001 [44]	11	3
Trost et al. 2000 [45]	1	4
Eliez et al. 2001 [46]	9	9
Vittorini et al. 2001 [47]	2	6
Chung et al. 2001 [48]	10	5
Saitta et al. 2004 [6]	35	30
Baumer et al. 2004 [33]	11	9
Our data	7	3
TOTAL	186 (56,3%)	144 (43,7%)

It has been proposed that LCR22-4 replicates later than LCR22-2 in chromosomes of maternal origin and that this favors misalignments between LCR22-2 and LCR22-4 leading to NAHR causing 22q11.2 deletions [33]. This model predicts that the remaining centromeric part of LCR22-2 in the deIeted chromosome would be predominantly of grandmaternal origin. In our case we determined that the centromeric region had a grandmaternal origin in 4 of the observed AHR events within LCR22-2, while 2 cases were not informative (data not shown). This observation seems to support an influence of late replication of the maternal chromosome in AHR events, as well as NAHR events. Nevertheless, when we analyzed three families that had suffered a NAHR event and a deletion, and contrary to previous results, only one had the centromeric portion of grandmaternal origin (data not shown). Futhermore, although 90% of the deletion events have their endpoints within LCR22-2 and 4, we find that the second highest AHR rate within an LCR22 is in LCR22-3a and not in LCR22-4. This observation argues against a direct and simple link between high AHR and NAHR levels. Other potential factors that might influence the participation of an LCR22 in a NAHR event may be, for example, the specific organization and content of each LCR (tandem versus inverted repeats), as well as the size and distance between the participating LCR22s.

The genomic diseases, Charcot-Marie-Tooth disease type 1A (CMT1A, MIM 118220) and hereditary neuropathy with liability to pressure palsies (HNPP, MIM 161500) are caused by LCR-mediated duplications and deletions in 17p12. *de novo *CMT1A duplication occurs 10 times more frequently in male than in female gametogenesis and AHR frequencies in the CMT1A/HNPP region indicate that the frequency is low in males but high in females [13]. Moreover, a similar observation was made for the genomic region that undergoes deletions causing the Smith-Magenis syndrome (17p11.2, SMS; MIM 182290) where a low rate of recombination was found in both sexes [14]. These observations led to the hypothesis that such reduced recombination may increase unequal crossing over by favoring misalignments [1].

Our study shows that LCR22s implicated in deletions and duplications are sites of frequent meiotic recombination (AHR) and that average recombination in the 22q11.2 region is similar to the chromosome average. Thus, aberrant recombination leading to 22q11.2 deletion syndrome can't be explained exclusively under a hypothesis of low regional AHR rates. This may reflect that the mechanisms causing NAHR in the 22q11.2 region and those in the regions causing SMS and CMT1A-HNPP may be different; or that available maps of the the 17p11-12 region do not have the resolution to determine AHR rates within the region's LCRs. Comprehensive high resolution maps of the 17p11-12 region may help solve this question.

In addition, we also show that AHR events within the 22q11.2 region cluster in families. If a concordance between AHR and NAHR exists, we would expect families with high AHR levels to be more prone to deletions and duplications. Although we would need a large number of multiple generation pedigrees to prove this, we describe here a family with two female crossovers within LCR22-2: in one generation it was an AHR event and in the next generation a NAHR event that resulted in a 22q11.2 deletion. We have not found differences in allelic frequencies of markers within 22q11.2 between individuals which show AHR and those that do not. However, it has been seen that the genetic background outside of a particular region influences recombination rates and breakpoint location [17,34]. Thus, it would not be surprising that the overall genetic background may have an effect on a slightly larger susceptibility to suffer rearrangements if NAHR and AHR are mechanistically related. If that is the case, the observed familial differences in recombination rates within the 22q11.2 region would have obvious health-related implications and certain families may have a slightly larger risk to suffer a 22q11.2 deletion. However, because the genetic or molecular factors that make 22q11.2 "hotter" for recombination are unknown the quantification of such risk is impossible right now.

In any case, further characterization of recombination in the 22q11.2 region should provide more information on a potential relation between NAHR and AHR active regions. However, high throughput studies that could clarify if AHR and NAHR show the same breakpoint locations are hampered by the recombinational and genomic traits of the 22q11.2 region. On one hand, LCR22s are large (300–400 kb) highly identical structures (>98% identity) making it very difficult to identify reliable polymorphisms that would allow localization of AHR breakpoints. And on the other hand, some of the most active LCR22s in NAHR and AHR may have higher female than male recombination rates, and thus sperm typing may not be the ideal technique to characterize AHR within these repeats.

## Conclusion

We present a high resolution recombination map of the 22q11.2 region based on pedigree data. Our map allows the location of recombination breakpoints with a high resolution (potential recombination hotspots), and this approach has led to the identification of 5 breakpoint segments of 50 kb or less (8.6 kb the smallest), that coincide with historical hotspots. It has been suggested that aberrant recombination leading to deletion (and duplication) is caused by low rates of Allelic Homologous Recombination (AHR) within the affected region [1]. Our study shows that LCR22s implicated in deletions and duplications are sites of frequent meiotic recombination (AHR) and that average recombination in the 22q11.2 region is similar to the chromosome average. Thus, aberrant recombination leading to 22q11.2 deletion syndrome can't be explained exclusively under a hypothesis of low AHR rates. In addition, we find recombination events in the 22q11.2 region to cluster within families. Within this context, the same chromosome recombines twice in one family; first by AHR and in the next generation by NAHR resulting in an individual affected with the del22q11.2 syndrome.

## Competing interests

The author(s) declare that they have no competing interests.

## Authors' contributions

LTJ carried out the molecular genetic studies, participated in the sequence analysis and in the draft of the manuscript. JR carried out clinical characterization of patients, data analysis and drafting of the manuscript. MST participated in the construction of the HapMap based recombination map and in the statistical tests. JF carried out the construction of the HapMap based recombination map and the statistical tests and participated in the drafting of the manuscript. DHS conceived the study, participated in the molecular genetic studies, analysed the data and wrote the manuscript. All authors read and approved the final manuscript.

## Pre-publication history

The pre-publication history for this paper can be accessed here:



## Supplementary Material

Additional File 1 Markers used for the construction of the pedigree linkage map. The table depicts the markers used for the construction of the pedigree-based recombination map. It depicts the name of the marker, its position on the chromosome, PCR product size, amplification primers, type of repeat and observed heterozigosity in our study. Click here for file
