# Supplementary material for: Analysis of meiotic recombination in 22q11.2, a region that frequently undergoes deletions and duplications
Source: BMC Med Genet. 2007 Apr 2;8:14. doi: 10.1186/1471-2350-8-14 (PMC1855045; doi:10.1186/1471-2350-8-14)
Supplement: Additional File 1 — Markers used for the construction of the pedigree linkage map. The table depicts the markers used for the construction of the pedigree-based recombination map. It depicts the name of the marker, its position on the chromosome, PCR product size, amplification primers, type of repeat and observed heterozigosity in our study. [file 1471-2350-8-14-S1.doc]

**ADDITIONAL FILE 1**

**Markers used for the construction of the pedigree linkage map**

| MARKER^1^ | POSITION | SIZE | PRIMER F | PRIMER R | REPEAT | Obs Het^2^ |
| --- | --- | --- | --- | --- | --- | --- |
| D22S420 | 16233835 | 154 | tgttctacactgaaaattctgacgg | gagggcgttatccatgacc | (GT)n | 0,77 |
| CATCH 5 | 16824839 | 116 | tatggattcacagtcttggccaac | tgtcagtgggatccccatgc | (CA)n | 0,45 |
| CATCH53 | 16901536 | 159 | acacccgtggcatggggcattc | cgtactttctatcaaacccgtgg | (GT)n | 0,68 |
| CATCH52 | 16946810 | 125 | tggtacattttcgggggttg | tgagctgagatcacgccaccctg | (TTTG)n | 0,16 |
| D22S427 | 16965871 | 96 | tgctgttttgtagagtgtttagac | aaatacggctgggcac | (CA)n | 0,63 |
| D22S1638 | 17369529 | 93 | gacaacagcaaattgcacatt | tcacgccactaccctccag | (GT)n | 0,64 |
| CATCH 4 | 17384345 | 80 | aaaaaattcagcaaggggtg | agtattctgtctatggcttgc | (GT)n | nd |
| CATCH 6 | 17484643 | 159 | cgtcttggtatttccaagcagc | ggaggacaagcttgccaggac | (CT)n | 0,33 |
| 22K48-2 | 17781918 | 142 | cggtgtgaatctataatcatctcg | acctctggtgtttggatatttacc | (CA)n | 0,51 |
| D22S1648 | 17781960 | 152 | agttgtcagatgcctaagaga | cagatgcttcaggagaagtg | (GT)n | 0,2 |
| D22S941 | 17784153 | 224 | caggttacaaagtacattaactt | accagctccaaccatttcttg | (CA)n | 0,51 |
| CATCH 48 | 17951980 | 200 | gatccttactgagaggaatgttgtg | agggtgatgaaaaggttctgg | (TA)n | 0,72 |
| D22S944 | 17984960 | 158 | catgtgaaagatgctacttcc | atggggaggagcatgggat | (GT)n | 0,48 |
| D22S1623 | 18022944 | 140 | cacaactcctgggctcaagct | acgtaaatctcataccatgtaaa | (CA)n | 0,36 |
| CATCH 22 | 18023379 | 178 | agtaggcaggggccataagg | cactgcactccagcttgggtg | (CTTC)n | 0,8 |
| CATCH 7 | 18122873 | 227 | cagcagcctgagcagcctgg | ccccggggccctcgggctcg | (CCG)n | nd |
| CATCH 23 | 18314831 | 221 | tgtgacagagtgacagcccgtctc | aggcccaccgtggcagcagc | (AAAT)n | 0,86 |
| CATCH 19 | 18684663 | 194 | gcacgttctgcacatgtacccc | tggcccaggagtccccatgc | (TA)n(CA)n | 0,45 |
| D22S264 | 19097786 | 190 | attaactcataaaggagccc | ggaatacctctggtggggtg | (CA)n | 0,8 |
| CATCH 45 | 19099847 | 167 | tgtttctctgtgtctggtggaag | atgtagacacagatatgcaccctgc | (GT)n | 0,42 |
| CATCH 10 | 19116496 | 129 | cacggtgtccggcagcatcc | cgcctcatggagggcgcagg | (CAG)n | nd |
| CATCH 20 | 19245111 | 161 | agatgagtgataaccgagtgc | tgaattggtggctgggcctg | (CAG)n | 0,75 |
| MID 1899 | 19255477 | 82 | tggaatctgctggtagaaag | acagccacacaacctaattc | SNP | 0,45 |
| MID 1900 | 19266719 | 145 | tacactgctgaagtgtggtg | tgactttcagagacagggaa | SNP | 0,47 |
| CATCH 42 | 19449222 | 98 | tgctatgttgcccaagctgg | actgtgtgtgtctctgtgtccagc | (CA)n | 0,8 |
| CATCH 41 | 19450208 | 179 | ctgttaccactaaatgggaaaatg | tgggtgacagagtgagactctg | (TTTA)n | nd |
| CATCH 11 | 19469542 | 194 | agccaaaatcacgccactgc | ctttatggttaccccttggacc | (CA)n | 0,62 |
| D22S311 | 19503575 | 262 | tttttgtatttttagtagagacgg | gctagtgtgagataacgaagcc | (CA)n | 0,8 |
| MID 1909 | 19542063 | 140 | tgggaacagaggagtatgag | aatctgtcatgtgttggcat | SNP | 0,48 |
| CATCH 12 | 19566292 | 116 | aaaggcagaaagcagccctg | gggtgaagagtaaagccttttcc | (CA)n | nd |
| CATCH 13 | 19567154 | 139 | cgtttggtggaggagggtcc | taacactgatcctttctgggagg | (CA)n | 0,66 |
| CATCH 14 | 19595454 | 213 | gaaggaaaggaaagggaaagg | ggccccgaggcttcccctgc | (AGAA)n | 0,87 |
| CATCH 39 | 19640114 | 110 | cactgcactccagcctgggag | caggctctgggatagccagtc | (TAAA)n | nd |
| MID 168 | 19677582 | 160 | gctggggaggatatgtca | gatttggaggaggcaacagagac | SNP | 0,46 |
| D22S1709 | 19735440 | 110 | cacttcagcaagaacagcaga | ctcttccaagttcagtgctct | (CA)n | 0,53 |
| CATCH 38 | 19745025 | 150 | gtacctgtagaattaccagtgctgg | ctccagcctgggcaacagagc | (ATTT)n | 0,52 |
| CATCH 37 | 19759833 | 232 | tggccgctgtgggctgcatg | gtgcatgccatcatgcccag | (CA)n | 0,47 |
| CATCH 36 | 20126412 | 110 | aatgccggggccgtcggtac | aacatctgcccatgcatacgg | (GT)n | 0,27 |
| CATCH 35 | 20341833 | 150 | ctgtactccagcctgggcaac | gtaaatgatgttccacccgtttg | (TAA)n | 0,57 |
| D22S446bis | 20343665 | 153 | ccaaggcaggcgaatcagg | tgtctggatgggcgtggtgg | (CA)n | 0,81 |
| CATCH 51 | 20418743 | 158 | actttcggaggctgaggcaggtgg | cctgggactacaggcgtgtgg | (GT)n | 0,8 |
| CATCH 33bis | 20510610 | 209 | cctggtcccagatacagtgc | gggtaatgggtggagattgatg | (GT)n | 0,71 |
| D22S539 | 20582334 | 124 | cattatggctgtaggctgta | cataccaatgcaatatgaa | (CA)n | 0,59 |
| CATCH 31 | 20677176 | 250 | gaacacacaggcccgctgttcc | caacaaaaatacccatgatacagctc | (GT)n(GA)n | nd |
| CATCH 25 | 20703346 | 177 | ttgtcaaggtcacaggcatc | gtggcattgtatgctgagatag | (GT)n | nd |
| CATCH 24 | 20703532 | 150 | tttacccagggttgggataggtgg | ttctccatggtaacgctaaaacc | (GT)n | nd |
| CATCH 26 | 20706255 | 174 | gcatatcaaaagctaagttatagc | gttccttcttttggttgaag | (CA)n(TA)n | 0,66 |
| CATCH 30 | 20740881 | 125 | gtctgctgacctgcatactc | ccaagggaactggaagcagg | (GT)n | 0,5 |
| CATCH 29 | 20818622 | 140 | ttagatactctgatgagccagc | ccctcaaatctttcagaatcag | (GT)n | nd |
| D22S308 | 20830724 | 196 | tcctgcaacagcactagacc | gctaaaggaaaaggaggcatc | (GT)n | 0,45 |
| CATCH 15 | 20851310 | 216 | taggcaggaatgggtcctg | ttcaacctgagggtggctgg | (CT)n(CA)n | 0,62 |
| D22S306 | 20887523 | 103 | ctctttcgctggaacatcaaa | ggtccagactgtataaatggc | (CA)n | 0,52 |
| CATCH 16 | 20896124 | 109 | gcagtaagatgatgactccggacc | atatggtaacacacatgcgtgtgc | (TG)n(CG)n | 0,83 |
| CATCH 28 | 20929869 | 185 | acctgcaccatgcttcctgc | gtaatatagcctaaacataaagtac | (TA)n | 0,5 |
| CATCH 17 | 21020043 | 161 | ccttacttgtatcctaggccagc | tgatgctgcagggcttggac | (TC)n(TG)n | nd |
| CATCH 27 | 21238686 | 247 | tgttccagcctgggcgacag | cctggtattgcctttccag | (GAAA)n | nd |
| CATCH 18 | 21256327 | 184 | gaagatagcccaccttgctgg | cctgagatattagggcaaacagg | (TG)n | 0,83 |
| D22S686 | 21393070 | 180 | ttgattacagagtggctctgg | taagccctgttagcaccact | (GGAA)n | 0,8 |
| D22S425 | 21407029 | 197 | tgcacaaggagacaactctg | tcatgccccataactcagg | (CA)n | 0,62 |
| D22S303 | 21599366 | 220 | aggacctcagactggtcagtc | ctcccatgagaaggtacactcc | (CA)n | 0,65 |
| D22S257 | 21892983 | 125 | agctgatacaccacttctga | gacagaaatatccttcccat | (TG)n | nd |
| D22S1174 | 2281304 | 214 | gaatcactaggggccttca | tgaggctatgtggcccag | (TG)n | 0,74 |

**^1^** Primers for the CATCH markers were developed for this study; other primers were taken from public databases.

**^2^** nd= not determined
